# Autism Spectrum Disorders: Multiple Routes to, and Multiple Consequences of, Abnormal Synaptic Function and Connectivity

**DOI:** 10.1177/1073858420921378

**Published:** 2020-05-22

**Authors:** Liam Carroll, Sven Braeutigam, John M. Dawes, Zeljka Krsnik, Ivica Kostovic, Ester Coutinho, Jennifer M. Dewing, Christopher A. Horton, Diego Gomez-Nicola, David A. Menassa

**Affiliations:** 1 Nuffield Department of Clinical Neurosciences, University of Oxford, Oxford, Oxfordshire, UK; 2 Oxford Centre for Human Brain Activity, Wellcome Centre for Integrative Neuroimaging, Department of Psychiatry, University of Oxford, Oxford, Oxfordshire, UK; 3 Croatian Institute for Brain Research, Centre of Research Excellence for Basic, Clinical and Translational Neuroscience, University of Zagreb School of Medicine, Zagreb, Croatia; 4 Maurice Wohl Clinical Neuroscience Institute, King’s College London, London, UK; 5 Faculty of Medicine, University of Southampton, Southampton, Hampshire, UK; 6 Sir William Dunn School of Pathology, University of Oxford, Oxford, Oxfordshire, UK; 7 Biological Sciences, Faculty of Environmental and Life Sciences, University of Southampton, Southampton, UK

**Keywords:** autism spectrum disorders, synaptic dysfunction, connectivity, neurodevelopment, phenotypic specificity, maternal immune activation, pain sensitivity, synaptic plasticity

## Abstract

Autism spectrum disorders (ASDs) are a heterogeneous group of neurodevelopmental disorders of genetic and environmental etiologies. Some ASD cases are syndromic: associated with clinically defined patterns of somatic abnormalities and a neurobehavioral phenotype (e.g., Fragile X syndrome). Many cases, however, are idiopathic or non-syndromic. Such disorders present themselves during the early postnatal period when language, speech, and personality start to develop. ASDs manifest by deficits in social communication and interaction, restricted and repetitive patterns of behavior across multiple contexts, sensory abnormalities across multiple modalities and comorbidities, such as epilepsy among many others. ASDs are disorders of connectivity, as synaptic dysfunction is common to both syndromic and idiopathic forms. While multiple theories have been proposed, particularly in idiopathic ASDs, none address why certain brain areas (e.g., frontotemporal) appear more vulnerable than others or identify factors that may affect phenotypic specificity. In this hypothesis article, we identify possible routes leading to, and the consequences of, altered connectivity and review the evidence of central and peripheral synaptic dysfunction in ASDs. We postulate that phenotypic specificity could arise from aberrant experience-dependent plasticity mechanisms in frontal brain areas and peripheral sensory networks and propose why the vulnerability of these areas could be part of a model to unify preexisting pathophysiological theories.

## Introduction

Autism spectrum disorders (ASD) is an umbrella term for disorders characterized by impairments in social interaction and communication as well as stereotypical behaviozrs of variable severity according to the *Diagnostic and Statistical Manual of Mental Disorders*, Fifth Edition (*DSM-5*) (American Psychiatric Association 2013). In addition to core symptoms and on average, about 25% of individuals have a clinical diagnosis of epilepsy (Muñoz-Yunta and others 2008; Spence and Schneider 2009) and have sensory abnormalities in multiple domains including somatosensation, vision, and olfaction (Cascio 2010; Menassa and others 2017; Menassa and others 2018). Twin studies indicate a significant genetic basis for these disorders with pairwise concordance for ASD varying between 60% and 95% in monozygotic twins versus 0% to 30% in dizygotic twins (Bailey and others 1995; Rosenberg and others 2009; Steffenburg and others 1989). A total of 5% to 15% of affected individuals possess an identifiable Mendelian condition corresponding to a syndromic gene disorder (Woodbury-Smith and Scherer 2018), with a significant proportion of sporadic and inherited ASDs resulting from dominantly acting *de novo* mutations (Zhao and others 2007). In a small number of cases, altered neurodevelopment, resulting in ASD-like symptomatology, has been attributed to maternal immune activation (MIA) (Bilbo and others 2018; Brown and Meyer 2018) or the maternal transfer of antibodies to the fetus (Coutinho and others 2017b; Dalton and others 2003; Dalton and others 2006), though it is not clear how phenotypic specificity arises here. Most cases of ASD are of unknown etiology. Nonetheless, despite their genetically and environmentally heterogeneous nature (Betancur 2011), ASDs converge on a shared symptomatology, suggesting that common molecular pathways may be dysregulated. A unifying theory that links such postulates and relates them to the connectivity patterns and synaptic abnormalities associated with ASD and addresses ASD-phenotypic specificity, is otherwise lacking. Such a theory may enable greater understanding of the relationship between genetic synaptopathies and ASD and inform novel therapeutic approaches.

The seminal study by Hubel and Wiesel demonstrated that, early in life, monocular deprivation in the dominant eye in a kitten shifts this dominance to the non-deprived eye (Wiesel and Hubel 1963). From this came the central role for experience-dependent synaptic plasticity in the development of neural circuits. Indeed, many of the genes mutated in ASD are crucial components of experience-dependent signaling processes that regulate synaptic plasticity (Table 1). While the genetic contributions to idiopathic ASD are heterogeneous and largely unknown, syndromic forms of ASD provide an invaluable tool to gain insight into the convergent molecular pathophysiology of ASD. In the following section, we identify the critical periods during human neurodevelopment and the postnatal age where synaptic dysfunction is likely to occur and contribute to ASD symptomatology. Next, we review the role of the immune system and microglia in altering synaptic circuits in ASD and present recent evidence on the outside-in theory of ASD arguing for a pivotal role for primary sensory neurons in some of the observed symptomatology. We then proceed to identify the most recent mechanisms by which brain connectivity is altered in ASD and conclude by proposing a model unifying existing ASD theories.

**Table 1.** Neurophysiological Roles of Autism Spectrum Disorders (ASD)–Linked Genes.

Gene	Protein Function	Disease Association	Main Role in Synapses	Syndromic Model
*MECP2*	Control of gene expression	Syndromic ASD mutation Rett syndrome	↑ Excitatory transmission ↑ Synaptic plasticity	Primate model (Liu and others 2016) Impaired social behavior ↑ S/RBs
*TSC1/2*	mTOR signaling antagonist	Syndromic ASD mutation Tuberous sclerosis	↑ Dendritic spine density ↑ Synaptic plasticity (LTP/LTD)	Mouse model (Chévere-Torres and others 2012) ↓ Social behavior
*CACNA1C*	Encodes α1-subunit of voltage-dependent Ca^2+^ channel	Syndromic ASD mutation Timothy syndrome	↑ Activity-dependent dendrite neurotransmission ↑ Synaptic plasticity (LTP)	Mouse model (Bader and others 2011) Impaired social behavior ↑ S/RBs ↓ USVs
*SHANK1*	Molecular scaffold in excitatory synapses	Rare single gene mutation linked to ASD	↑ Basal excitatory synaptic transmission	Mouse model (Sungur and others 2014) ↓ Social behavior ↑ S/RBs ↓ USVs
*SHANK2*	Molecular scaffold in excitatory synapses	Syndromic ASD mutation	↑ Synapse formation ↑ Synaptic plasticity (LTP)	Mouse model (Schmeisser and others 2012) Impaired social behavior ↑ S/RBs ↓ USVs
*SHANK3*	Molecular scaffold in excitatory synapses	Syndromic ASD mutation Phenlan-McDermid syndrome	↑ Synapse formation ↑ Synaptic plasticity	Mouse model (Peça and others 2011) ↓ Social behavior ↑ S/RBs
*NRXN1*	Cell adhesion molecule in the nervous system	Syndromic ASD mutation Pitt-Hopkins-like syndrome 2	↑ Ca^2+^-driven neurotransmission ↑ Synaptic plasticity (LTP)	Mouse model (Etherton and others 2009) No difference in social behavior ↑ S/RBs
*NLGN3*	Neural cell surface molecule	Single gene mutation linked to ASD	↑ Synapse formation ↑ Synaptic plasticity (LTP)	Rat model (Hamilton and others 2014) ↓ Social behavior
*NF1*	Negative regulator of cell proliferation	Syndromic ASD mutation	Regulates GABA release ↑ Synaptic plasticity	Mouse model (Costa and others 2001) Impaired social behavior N/A S/RBs N/A USVs
*PTEN*	Regulator of PI3K signaling	Syndromic ASD mutation Cowden syndrome	↓ Spine density ↑ Synaptic plasticity (LTD)	Mouse model (Lugo and others 2014) Impaired social behavior ↑ S/RBs No difference in USVs
*CNTNAP2*	Synaptic adhesion molecule	Syndromic ASD mutation Cortical dysplasia-focal epilepsy syndrome	↓ GABAergic interneurons ↓ Neuronal synchrony	Mouse model (Peñagarikano and others 2011) ↓ Social behavior

LTD = long-term depression; LTP = long-term potentiation; N/A = not applicable/not tested; S/RBs = stereotypical repetitive behaviors; USVs = ultrasonic vocalizations.

## Neurodevelopmental and Postnatal Circuitry Disturbance in ASD Development

The way by which generalized synaptic dysfunction in ASD might lead to patterns of cortical connectivity and specific behavioral impairments, while preserving or even enhancing other behaviors (Mottron and others 2006) remains an open and important question. Core autistic behaviors may be explained by developmental disconnections between higher order association areas (Just and others 2004; Just and others 2007; Ozonoff and others 1991; Perez Velazquez and others 2009) such as the dorsolateral prefrontal regions and anterior cingulate cortex and other cortical areas. Considering a prospective genetic and environmental etiology, it is important to determine when an initial disturbance of the circuitry develops, and which components of cortical circuitry and functional networks are mostly affected. Different components of cortical connectivity develop sequentially, but in a partly overlapping manner, from the late embryonic period through to young adulthood (Petanjek and others 2011). Furthermore, these periods of rapid growth may be particularly vulnerable to genetic insults (Kostović and others 2014). Recent progress in genetic, genomic and transcriptomic ASD research has elucidated various coding and non-coding variances and co-expression networks, which show spatiotemporal preferences and may cause abnormalities of the presynaptic and postsynaptic molecular assembly of synapses (Gandal and others 2018; Geschwind 2009; Koopmans and others 2019; Molnár and others 2019; Sestan and State 2018; Zhu and others 2018). In particular, a whole range of presynaptic and postsynaptic proteins may be affected due to alteration of ASD-risk genes (Table 1). It is important to note here that ASD risk genes for the pre- and postsynaptic circuitry does not necessarily mean that synapses are the only point of failure in ASD. SNARE complex proteins mediate the fusion of presynaptic vesicles with the plasma membrane and intracellular vesicle growth cone and leading filopodia external membranes, thereby providing a mechanism for directed growth and migration. For example, neurexins have non-synaptic roles during development (Harkin and others 2019; Tsaneva-Atanasova and others 2009). Although we specifically focus on synaptic dysfunction in this review, dysregulation of axonal growth and pathfinding can play a role in the etiology of ASD, as candidate ASD-susceptibility genes impinge upon these processes (McFadden and Minshew 2013). Furthermore, subtle deficits in these processes could play a part in the failure of long-distance pathway formation in ASD.

### The Prenatal Period

The analysis of gene expression during the mid-fetal period indicates inner cortical plate (CP) projection neurons as a prospective target in ASD (Sestan and State 2018; Willsey and others 2013). Recent work also suggests that, in comparison with typical development, differentially expressed genes in ASD are down-regulated in layer 2/3 excitatory neurons and upregulated in protoplasmic astrocytes and microglia (Velmeshev and others 2019). Both observations are in accordance with previous Golgi studies showing that during late mid-gestation, the phenotype of cortical projection neurons is rapidly developing (Marin-Padilla 1970; Mrzljak and others 1988). However, late mid-gestational peak expression of synapse-development genes occurs after initial synaptogenesis during early fetal life (Huttenlocher and Dabholkar 1997; Kang and others 2011; Kostović and Rakic 1990; Kostović and Krmpotić 1976; Kostović and others 1989).

**Figure 1.**
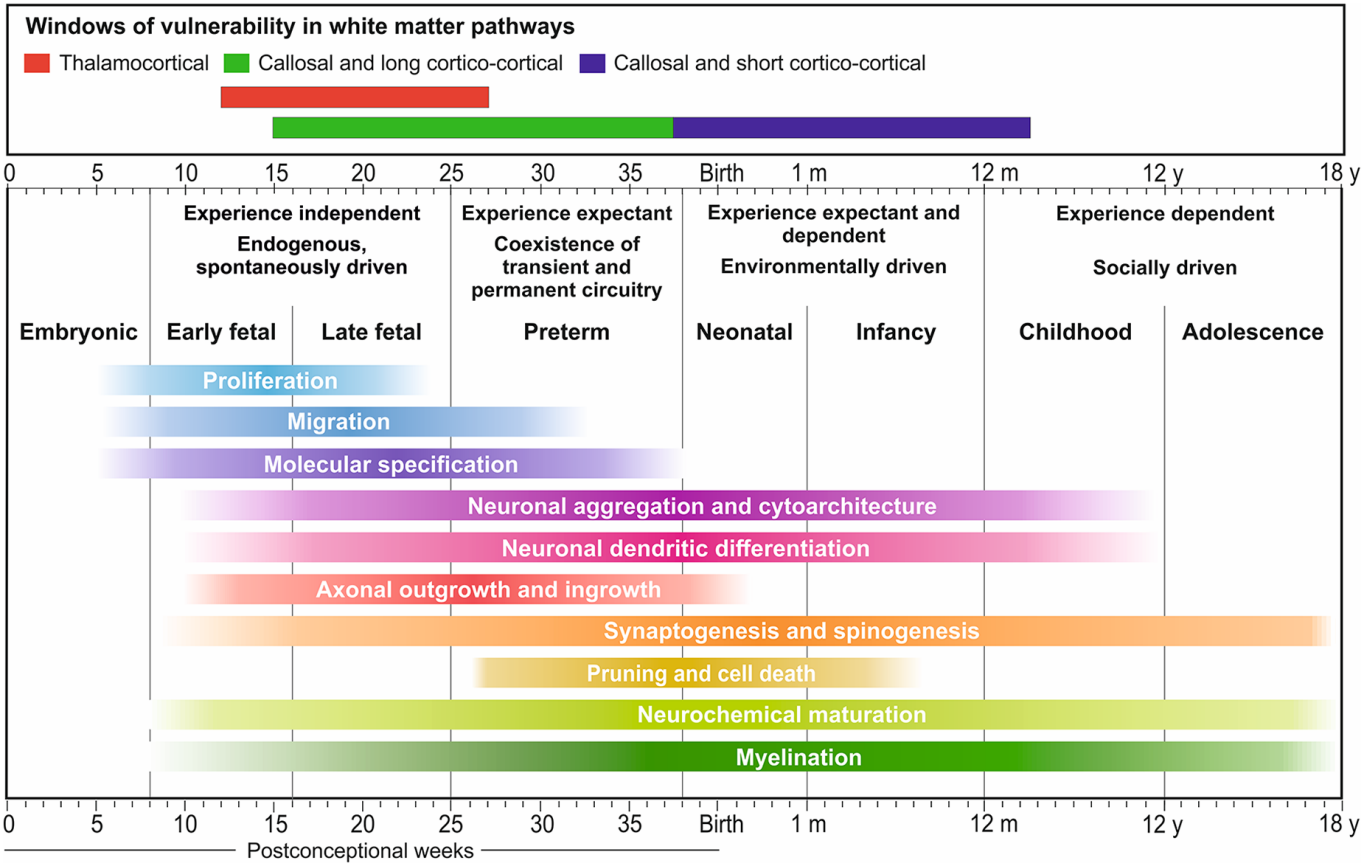
Timing of human neurogenetic events. These events can be affected by genetic and environmental factors during prenatal and postnatal periods, leading to abnormal cortical organization and complex cognitive and behavioral deficits in humans. The critical period for the interaction of presynaptic axons and postsynaptic neurons during initial synaptogenesis and the formation of cortical circuitry begins at the early fetal period and shows prolonged periods of prospective vulnerability: during the early preterm for thalamocortical connections (red bar), and during the late preterm for callosal and long cortico-cortical connections (blue), which may correspond to a 1st hit event. However, since short cortico-cortical pathways continue during infancy and early childhood (purple), peaking at around 2 years for associative cortex for example, we may expect vulnerability that corresponds to a second hit in the pathogenesis of circuitry relating to ASD. Modified with permission from Kostović I, Judaš M. (2015). Embryonic and fetal development of the human cerebral cortex in brain mapping: an encyclopaedic reference; volume 2: anatomy and physiology, systems, Elsevier.

During mid-gestation, synapses are found in transitional cortical areas called the subplate (SP) (which contains future interstitial gyral white matter neurons) and the marginal zone (MZ) (future layer I), whereas at the transition between mid and late mid-gestation (after 24 postconceptional weeks [pcw]) synapses develop rapidly in the CP (Kostović and Rakic 1990; Kostović and others 1989; Kostović and others 2019a; Molliver and others 1973). The SP is where the earliest connectivity and functional activity begin in the developing cortex (Moore and others 2009; Moore and others 2011). Fetal circuitry is then spontaneous and endogenous (Friauf and Shatz 1991; Kanold and Luhmann 2010; Kostović and Judaš 2015) (Fig. 1). During this window, it is difficult to ascertain whether environmental influences affect synapse development. Furthermore, more evidence is needed to concur on whether SP and MZ synapses during mid-gestation (Kostović and Rakic 1990; Molliver and others 1973) participate in spontaneous activity or whether they are silent (Meng and others 2014). Using *in vitro* patch- clamping studies in human SP neurons during mid-gestation, synaptic potentials could be elicited (Moore and others 2009; Moore and others 2011). SP neurons may be activated by the stimulation of thalamic axons before CP neurons (Allendoerfer and Shatz 1994; Friauf and Shatz 1991). This finding corresponds to observations in humans, where the first synapses within the CP of the somatosensory and visual cortices are seen only after 23 pcw (Kostović and Rakic 1990; Molliver and others 1973). Thus, this period may be described as sensory-expectant, and one cannot exclude activity influences of afferents from the thalamus and the basal forebrain (Kostović and Judaš 2002, 2010). Indeed, recent evidence suggests that the primate SP receives thalamocortical innervation much earlier than previously thought (Alzu’bi and others 2019). Furthermore, arealization, which is the process of innervation of cortical areas by specific thalamic nuclei, has been proposed as core to the eventual establishment of long-range connectivity (Moreno-Juan and others 2017).

After the 24th pcw and during the entire late fetal period, the situation changes and cortical responses are evoked by peripheral stimulation (Fitzgerald 2005; Khazipov and Luhmann 2006; Kostović and Judaš 2010; Leroy and others 2011). Thus, a sensory-expectant form of transient cortical circuitry gradually shifts into a sensory-evoked cortical circuitry (Kostović and Judaš 2015). Alongside increasing activity in the CP, transient activity still remains within the SP, and this prolonged activity of both transient and permanent circuitries seems to be a salient feature of the human brain (Kostović and Judaš 2006). During this period, there is also intensive growth of callosal and long associative pathways (Huang and Vasung 2014; Huang and others 2006; Vasung and others 2017), at a time associated with a high expression level of a myriad of genes involved in synaptogenesis (Pletikos and others 2014).

Altogether, the above delineate the late mid-gestation and late gestation/preterm periods as critical periods for the initial interaction of converging ASD-risk genes, development of cortical pathways within the frontal, somatosensory, visual, and limbic areas as well as synaptic interactions within the thalamus, striatum, amygdala, and basal forebrain (Kostović and others 2019b). However, it remains unclear as to whether interactions with the environment in prematurely born neonates or interactions with external stimuli *in utero* prior to birth alter the development of circuitry during typical development. Experimental studies in primates suggest that environmental influences may change the structure of preexisting synapses, but not the total number of produced synapses (Bourgeois and others 1989) indicating that prenatal synaptogenesis in primates is genetically programmed and experience-independent.

There is currently no evidence to suggest that atypical functional networks found in ASD, such as the frontoparietal and salient/ventral attention networks, are selectively damaged during an initial insult. The functional networks involving the limbic structures are most likely the candidates for developmental disturbances, as synapses in the hippocampus and cingulate gyrus appear to develop at a faster rate than within the neocortex (Kostović and Krmpotić 1976; Kostović and others 1989).

The differences in timing and pace of synaptogenesis in the hippocampus, anterior cingulate gyrus (“limbic cortex”) and lateral fronto-parietal neocortex is significant for differential vulnerability of these cortical networks. If one of these networks is injured and the other is spared in intrauterine life, disconnectivity may ensue, changing further the development of synapses, because the timing of structural synaptic connectivity is essential for the further development of circuitry.

It is even less clear as to when and how, during postnatal development, cortical circuitry and synapses undergo structural and functional alteration, leading to the expression of ASD symptomatology. It follows that, if the first red-flag of ASD symptoms appears by 2 to 3 years of age, and the full spectrum of the disorder is visible later during childhood (Lord and others 2000), then the search for critical periods when abnormal circuitry develops must be concentrated on the first two years of postnatal life (Gao and others 2015; Kostović and others 2014; Pletikos and others 2014). During this time, there is a dramatic increase in synapse and spine production in cortical areas (Huttenlocher 1999; Petanjek and others 2011). Synapses develop and reach their plateau more rapidly in primary sensory areas, such as the primary visual cortex, than in associative frontal areas (Huttenlocher 1999). Parallel to the process of synaptogenesis, there is substantial growth of pyramidal neuron dendrites during the first 2 years of life, alongside a dormant period for layer III pyramidal neurons between 2.5 and 16 months (Petanjek and others 2011).

### The Postnatal Period

The postnatal period is characterized by the growth of short cortico-cortical pathways, which may contribute to a significant reorganization of cortical circuitry and synapse function (Kostović and others 2014). The development of whole brain functional architecture during the first two years shows significant changes in both within and between-network interactions (Gao and others 2015). The early increase in the connectivity of primary networks shows that partial connectivity decreases (Gilmore and others 2018). Higher order networks, which are topologically incomplete in neonates, show synchronization and connectivity increases during the first 2 years of life (Gao and others 2015). Similar developmental trends have been demonstrated in the emergence of the brain’s default networks which include the prefrontal, posterior cingulate/retrosplenial, inferior parietal, and hippocampal cortices. It seems that the main “hub” is the posterior cingulate/retrosplenial cortex, whilst the medial prefrontal cortex may represent a potential secondary hub, beginning from 1 year of life (Gao and others 2009).

During the early postnatal period, the cortex is environmentally driven and gradually becomes experience/sensory dependent. This phenomenon is best known from the study of the visual system (Maurer and others 1999). The background underlying such changes in connectivity is myelination, which is synchronized in order to provide balanced activity of remote cortical areas (Salami and others 2003) and may substantially participate in changes within different networks during the first and second years of life. The general indicator of developmental change within cortical organization is cortical thickness, which can be monitored in large cohorts of those with ASD and healthy controls (Khundrakpam and others 2017). Approaching the second half of the first year, social stimuli become more important and may modify socially driven cortico-cortical networks (Ciarrusta and others 2019).

All of the above suggest that developmental events may be disturbed during the first and second year of life, which may alter synaptic function of selected cortical circuitry, and this alteration may underlie a “second-hit” event in the developmental pathogenesis of ASD. Thus, an initial, first phase abnormality of synaptogenesis and differential vulnerability of limbic and lateral neocortical networks in prenatal life and subsequent developmental reorganization during the second postnatal phase may contribute to alterations of connectivity referred to as overconnectivity, disconnectivity, and hypoconnectivity (Ciarrusta and others 2019; Courchesne and others 2005; Geschwind 2009; Yerys and others 2019). According to (Picci and Scherf 2015), early perturbation of cortical connectivity may be considered as a “first-hit,” which sets up a neural circuitry that is “built to fail” in the face of a second-hit that occurs during late childhood.

## The Role of the Immune System in ASD Pathophysiology

*In utero* or early life exposure to an abnormal immune response is a known risk factor for ASD. This is supported by several lines of evidence from different fields, including epidemiology and immunology (Estes and McAllister 2015). This is the basis for the “immune theory” of ASD, which postulates that a genetically predisposed individual, if exposed to an immune system stressor (such as environmental toxins, infections, or maternal immune molecules) during the prenatal or early postnatal period, will have irreversible neural circuit changes (Gottfried and others 2015), which will eventually lead to behavioral symptoms.

Epidemiological evidence shows a link between exposure to infectious agents during pregnancy and an increased risk for neurodevelopmental disorders in the progeny (Knuesel and others 2014; Patterson 2002). This is supported by the “winter baby” phenomenon (Zerbo and others 2011), which describes an increased risk for ASD in children conceived in the colder months. There is, as yet, no clear association with a particular infectious agent; infections by a virus, bacterium, and parasite have all been linked to neurodevelopmental disorders, which likely indicates a common mechanism caused by maternal immune activation (MIA) and not the infection *per se*. It is postulated that this MIA *in utero* might result in chronic dysfunction in the progeny, since neuropathological studies had revealed the presence of markers of inflammation such as microglial activation in patients with ASD (Rodriguez and Kern 2011). Additionally, increased pro-inflammatory markers within the serum and cerebrospinal fluid (Ashwood and others 2011; Chez and others 2007; Zerbo and others 2014) have also been reported in ASD, which persist many years after disease diagnosis. Animal models support the link between maternal infections, MIA and structural and behavioral anomalies in the offspring. Existing models are based on maternal exposure to the infectious agent (e.g. human influenza virus), a viral mimetic (e.g., polyinosinic-polycytidilic acid [poly(I:C)] or bacterial mimetic (lipopolysaccharide [LPS]) or the immune mediators themselves (inflammatory cytokines) (Meyer and others 2009). Additionally, MIA is associated with defective microglial synaptic pruning whereby mouse progeny have autistic-like behaviors (Fernández de Cossío and others 2017). Microglia seem to be implicated directly or indirectly in the pathological mechanism shared between different causes of ASD. This is further supported by impaired functional connectivity and autistic-like behaviors in CX3CR1^−/−^ mice lacking responsive microglia (Zhan and others 2014), for example, though this could be due to two reasons: a transient decreased microglial density in postnatal development, or the fractalkine signaling pathway being responsible for the tagging of synapses.

A compelling hypothesis that microglia influence brain growth by regulating early postnatal neurogenesis and synaptogenesis by pruning mechanisms has gathered interest in recent years (Cunningham and others 2013; Paolicelli and others 2011; Shankle and others 1999). This raises the question as to whether microglial activity may be adversely affected by ASD-linked synaptic mutations, and whether this may result in deficient pruning of developing synaptic connections, leading to overconnectivity.

Microglia are present in the brain from early development, derived from erythro-myeloid progenitors (EMPs) originating in the yolk sac (YS) (Ginhoux and others 2010; Menassa and Gomez-Nicola 2018; Verney and others 2010). Early microglial progenitors seed the brain, named pre-macrophages (pMacs), expand and persist into adulthood to form the resident microglial population in the adult brain (Epelman and others 2014; Ginhoux and others 2010). Microglia develop in three steps (early, pre-, and adult microglia), in synchrony with brain development (Matcovitch-Natan and others 2016). From an initial limited number of infiltrating progenitors, the population expands rapidly to colonize all brain regions by birth. During postnatal development, microglial numbers continue to increase until postnatal day 14, to later undergo a selection phase before achieving the final adult densities (Askew and others 2017; Nikodemova and others 2015). This is based on rodent studies and is unknown in humans. Rodent studies indicate that monocyte infiltration and differentiation do not contribute to the postnatal microglial population, although it is still unclear if transient waves of monocyte infiltration could drive functional changes in brain development (Askew and others 2017). Altogether, our current understanding of microglial dynamics during embryonic and postnatal brain development supports an intimate bidirectional communication with the brain’s environment, as microglia can alter neuronal numbers and synaptic contacts, and neurons can influence microglial phenotypic specification (Askew and Gomez-Nicola 2017).

Various studies support the notion that synaptic contacts with microglia and microglial-mediated synaptic pruning are regulated by neural activity in an experience-dependent manner (Parkhurst and others 2013; Schafer and others 2012; Tremblay and others 2010). Furthermore, disturbance of experience-dependent plasticity mechanisms at a neuronal level by ASD-linked mutations impinges onto microglial pruning mechanisms, resulting in overconnectivity (Table 1).

Further evidence for the involvement of microglia in ASD etiology comes from the literature on the transfer of maternal pathogenic antibodies to the fetus. The first cohort study linking maternal antibodies to ASD dates back to the early 1990s where paternal lymphocyte epitopes in the sera of mothers of children with ASD were identified (Warren and others 1990). These findings were largely forgotten and studies of maternal-to-fetal transfer of serum from mothers of autistic or dyslexic children in mouse models were published a decade later (Dalton and others 2003; Vincent and others 2002). These demonstrated in the mouse offspring deficits in neuromotor coordination and cerebellar metabolite changes. Additionally, maternal serum bound the surface of Purkinje cell neurons suggesting a neuronal surface antigen. Since then, the presence of antibodies against fetal antigens in the sera of mothers of autistic children has been reported (Braunschweig and others 2012; Brimberg and others 2013; Piras and others 2014; Zimmerman and others 2007). More recently, case-control studies of gestational samples found that antibodies against CASPR2, a cell adhesion protein of the neurexin family, were frequent in mid-gestational sera from mothers of children with intellectual disability (Coutinho and others 2017a) or ASD (Brimberg and others 2016). Importantly, *in utero* exposure to CASPR2-antibodies, in a passive immunization maternal-to-fetal mouse model, led to irreversible abnormalities in the offspring manifested by deficits in social behaviors, cortical lamination abnormalities, increased activated microglial numbers correlating with a loss of glutamatergic synapses (Coutinho and others 2017b). These studies strongly support a causal link between maternal antibodies and neurodevelopmental deficits in the offspring and put tangible evidence behind the concept of maternal antibody-mediated neurodevelopmental disorders. Importantly, they hint toward an effect of pathogenic maternal antibodies in the process of microglia-dependent synaptic refinement, which could be the link between the immune system and neuronal circuit development. This is certainly an area to explore in future studies.

A prospective link between the immune system and the damage of associative circuitry may occur in cases of periventricular focal lesions in preterm infants, namely, infection and activation of the immune system, in combination with hypoxia-ischemia. This may damage associative periventricular pathways in areas of axonal crossroads, which show increased vulnerability (Kostović and others 2014). It was also shown that in a cohort of preterm infants there is a tendency of higher prevalence of ASD (Limperopoulos and others 2008). Hypoxic-ischemic factors may also disturb the activity of axonal guidance molecules in periventricular vulnerable areas leading to altered connectivity that can underlie ASD (McFadden and Minshew 2013).

## The Role of the Peripheral Nervous System in Synaptic Dysfunction in ASD

Altered somatosensation, such as hypersensitivity to touch or abnormal pain sensitivity, is common in people with ASD and assessment of sensory function is now part of the diagnostic criteria (Cascio 2010). The first step in normal somatosensation is activation of specialized sensory endings in the skin such as low-threshold mechanoreceptors and free nerve endings of nociceptors and thermoreceptors. These peripheral neurons have been somewhat overlooked in terms of providing an explanation for the sensory phenotype observed in those with ASD. Indeed, abnormal sensory responses in ASDs may arise purely from altered processing at the level of the central nervous system (CNS). However, recent preclinical studies point to dysfunction of the peripheral nervous system (PNS) as an important driver not only of abnormal sensation but also other core ASD-like behaviors.

**Figure 2.**
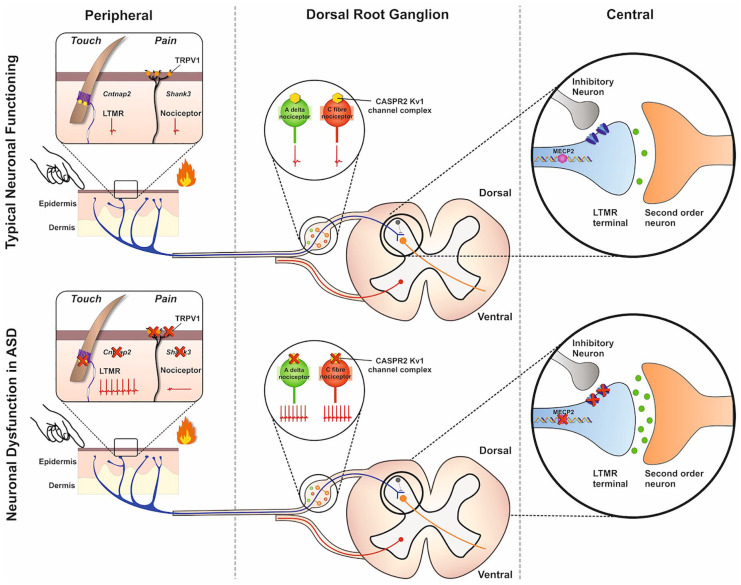
Dysfunction of primary sensory neurons in autism spectrum disorders (ASD). By genetically altering ASD-linked genes, several mouse models have been developed. Some of these models have shown phenotypic changes in somatosensation associated with primary sensory neuron dysfunction. This dysfunction is linked not only to the synapse (the central terminal of the dorsal horn of the spinal cord) of these neurons but also to other neuronal compartments (e.g., the peripheral terminal in the skin and the cell soma). Loss of Cntnap2 leads to hyperexcitability in d-hairs, a type of low threshold mechanoreceptor (LTMR), due to loss of Kv1 channel function. Loss of Shank3 reduces the functional expression of TRPV1, a transduction channel important in heat hyperalgesia, in nociceptors. Soma loss of Cntnap2 also impacts onto nociceptor function resulting in hyperexcitablility of Aδ and C fibers. At the level of the synapse, loss of MECP2 results in the down regulation of GABA receptors and loss of presynaptic inhibition on LTMRs leading to increased sensitivity to tactile stimuli.

In terms of expression, using recently created searchable transcriptional sequencing databases, it is interesting to note that many of ASD candidate genes, which are known to be important for synapse formation and function, (e.g., *NRXNs, NLGNs, SHANKs, FMR1, CNTNAP2* and *MECP2*, Table 1) (Guang and others 2018) are well- expressed by primary sensory neurons (Table 2). In line with this, a number of genetic ASD mouse models display abnormal sensory behavior alongside the more characteristic ASD-like behaviors such as anxiety and reduced sociability (Table 2). For instance, genetic mutations in *Mecp2, Frm1*, and *Shank3* all result in impairment of discriminate touch and hypersensitivity to tactile stimuli (Orefice and others 2016). Since these genetic models affect gene expression throughout the whole organism, it is difficult to untangle the contribution of the PNS and CNS. In an effort to tackle this issue, a recent study using conditional ablation models of Rett syndrome–linked *Mecp2* mutations (Orefice and others 2016), which are also associated with ASDs (Wen and others 2017), showed that specific ablation from primary sensory neurons resulted in the same sensory phenotype as global deletion. In contrast, specific deletion from CNS regions, such as the forebrain, did not impair sensory behavior when compared with littermate controls. These findings imply that loss of function of *Mecp2* in the PNS, and not the CNS, is the reason for altered sensation in this model of ASD. Remarkably, when *Mecp2* was introduced back into sensory neurons in the global *Mecp2*-mutant mouse, mechanosensation and other ASD-like behaviors, such as sociability, were normalized. These changes were linked to a decrease in the expression of the beta-3 subunit of the GABA_A_ receptor at the central terminal of low-threshold mechanosensitive neurons. This resulted in a hyperexcitable synapse due to loss of presynaptic inhibition and, as a consequence a loss of control on tactile sensory input (Note: Mutations in *GABRB3* are also strongly linked to ASD [Delahanty and others 2011] and specific deletion in primary sensory neurons, also causes ASD-like behaviors). These data therefore suggest that altered synaptic function of peripheral neurons, due to ASD candidate gene mutation, is not only important for abnormal responses to tactile stimuli, but their dysfunction may also drive other ASD-like behaviors whose origin has traditionally been considered CNS specific (Orefice and others 2016) (Fig. 2).

**Table 2.** Expression of Select Autism Spectrum Disorders (ASD)–Linked Genes in Primary Sensory Neurons and Somatosensation in Mouse Models.

ASD Gene	SFARI Score^a^	Expression by Primary Sensory Neurons^b^		Altered Somatosensation in Mouse
		Human	Mouse	
***MECP2***	2/S	Good	Good	Touch and pain (Orefice and others 2016)
***FMR1***	S	Good	Good	Touch (Orefice and others 2016)
***SHANK3***	1/S	Low	Good	Touch and heat pain (Han and others, 2016)
**SHANK2**	2	Low	Low	Mechanical and heat pain (Ko and others 2016)
***CNTNAP2***	2/S	Good	High	Mechanical and heat pain (Dawes and others 2018)
***GABRB3***	2	Good	High	Touch, mechanical and heat pain (Orefice and others 2016)
***CDH8***	4	Good	Good	Cold (Suzuki and others 2007)

a Score indicates how well associated the gene is with ASD. 1 (highest), 5 (hypothesized but untested), S (associated with a syndrome similar to ASD).

b Data taken from online database (Ray and others 2018). Low <10 TPM (transcripts per million), good 10 to 99 TPM, high >100 TPM. Expression levels are measured from whole lumbar dorsal root ganglion samples.

In terms of pain sensitivity, there are reports of ASD phenotypes with both hypo- and hyper-sensitivity (Cascio 2010; Vaughan and others 2019). *Shank3* knockout mice are a model of ASD (Zhou and others 2016) and show deficits in heat sensitivity (Han and others 2016). Indeed, Shank3 is highly expressed by primary sensory neurons, including nociceptors, particularly at the level of the presynaptic terminal in the superficial dorsal horn. Here, it interacts with Trpv1, an important protein in the transduction of noxious heat in the skin and also expressed at the central terminal. Using the Trpv1 specific algogen capsaicin, loss of Shank3 results in decreased synaptic transmission due to a loss of Trpv1 surface expression. These findings again point to disruption of synaptic function in primary sensory neurons as the substrate for ASD-like behaviors, in this case, pain insensitivity. This is further supported by the observation that nociceptor-specific removal of *Shank3*, but not from other parts of the nervous system, replicate the aberrant thermosensitivity phenotype (Han and others 2016)). Furthermore, genetic disruption of other ASD associated genes, such as *GABRB3* and *CNTNAP2*, result in ASD-like behaviors in mice (DeLorey and others 2008; Peñagarikano and others 2011), including pain (Dawes and others 2018; DeLorey and others 2011). Although expression of these genes is altered globally, isolation of primary sensory neurons from *Cntnap2* knockout mice, show that these neurons are dysfunctional. For example, compared to control neurons, disruption of *Cntnap2* results in hyperexcitability due to loss of surface Kv1 channels and provides an explanation for the pain hypersensitivity phenotype (Dawes and others 2018). This phenomenon was observed at the level of the cell body as well as nerve terminals in the skin. Such findings suggest that ASD-associated genes may disrupt primary sensory neuron function not only at the central terminal but also in other neuronal compartments.

Since these genes are well expressed by primary sensory neurons, their loss might result in altered structural development and hence, abnormal sensation. Although one study reports on lower epidermal nerve fiber density in ASD (Silva and Schalock 2016), there is a lack of other studies in this area. From ASD mouse models, primary sensory neurons are structurally normal in terms of skin innervation, subpopulation distribution and their central terminals (Dawes and others 2018; Han and others 2016; Orefice and others 2016). Instead, it seems that in terms of primary sensory neuron biology, ASD genes directly alter function at the level of the synapse and other neuronal compartments, to alter tactile sensitivity and pain, independently of neurodevelopmental mechanisms. In agreement with this idea, antibody-mediated disruption of the protein product of *Cntnap2* (CASPR2), or genetic ablation of *Mecp2*, from primary sensory neurons in adulthood result in the same altered sensory behaviour compared to when these genes are removed during development (Dawes and others 2018; Orefice and others 2016; Orefice and others 2019). However, abnormal sensory input might also have an important developmental role in shaping the wider symptomology of ASD. For example, studies investigating touch deprivation during development in humans and animal models show that lack of touch impacts onto cognitive behaviors (Ardiel and Rankin 2010; Cascio and others 2019). Given that touch can affect synapse formation in areas of the brain such as the prefrontal cortex (Kolb and others 2012), aberrant sensory input may be a key driver in altered synapse development in ASD patients. In line with this, removal of *Mecp2* or *Gabrb3* from primary sensory neurons in adulthood only affects tactile behavior, whereas removal from these neurons during development also causes anxiety and reduced social behavior and circuitry changes within the brain (Orefice and others 2019). Therefore, abnormal tactile experience-dependent synapse development may be a fundamental pathophysiological mechanism underlying ASD and the targeting of primary sensory neurons offers an alternative strategy for the treatment of core ASD behaviors.

## Evidence of Altered Connectivity in ASD

There is no doubt that a deeper understanding of abnormal synaptic connectivity, and by extension, structural connectivity defined by axonal pathways, is critical for the study of ASDs. Besides this so-called static connectivity, functional connectivity is beginning to play a major role in ASD research. Broadly speaking, functional connectivity refers to the dynamic and functionally unified relationship between brain areas regardless of apparent neuronal connections between the regions (Friston 2011). In neuroimaging applications, functional connectivity is typically defined as the possible causal correlation between neurophysiological events, quantified through some measure, where deviation from statistical independence of such events is assumed to indicate connectivity (Table 3).

**Table 3.** Brain Connectivity Patterns in Autism Spectrum Disorders (ASD).

ASD Clinical Cohort	Analysis Method	Behavioral Findings	Connectivity Findings	Definition of Connectivity Range
Tuberous sclerosis (n = 14; mean age: 9.3 years) (Peters and others 2013)	Resting-state EEG	N/A	↓ Long-range connectivity ↑ Local connectivity	Not defined
High functioning ASD (n = 10; mean age: 23.8 years) (Barttfeld and others 2011)	Resting state, eyes closed EEG	ASD severity related to ↑ short-range coherence and ↓ long-range coherence	↓ Long-range connectivity ↑ Local connectivity	Local connectivity defined as cortico-cortical connections between the same cortical areas. Long-distance connectivity defined as cortico-cortical connections between different functional areas
*NF1* mutation (n = 14; mean age: 12.49 years) (Loitfelder and others 2015)	Resting state fMRI	Relative connectivity levels correlated with parent reports of cognitive, social and behavioral functioning	↑ Frontofrontal connections ↑ Temporofrontal connections ↓ Left amygdala-PCC coupling	Not explicitly defined.
*NF1* mutation (n = 30; mean age: 27 years) (Tomson and others 2015)	Resting state fMRI	Differences in local connectivity were correlated with IQ and internalizing symptoms	↓ Long-range connectivity ↑ Local connectivity	Local connectivity appears to be defined as connections within visual networks. Long-range connectivity defined as anterior-posterior connectivity and within the DFN (default-mode network).
ASD diagnosed via ADI-R (n = 12; mean age: 26.5 years) (Kennedy and Courchesne 2008)	Resting state fMRI	N/A	↓ Long-range connectivity Regional abnormality in local connections	Local connectivity investigated in localized areas of the DFN. Long-distance connectivity defined as the DFN.
ASD including 11 with Asperger’s (n = 26; mean age: 26 years) (Shukla and others 2011)	Resting state DTI	N/A	↓ Long-range connectivity ↓ Local connectivity	Local connectivity defined as those <35 mm including subcortical U-fibers. Long-distance connectivity defined > 65 mm.
ASD including PDD-NOS, Asperger’s (n = 16, mean age: 57.5 years) (Sundaram and others 2008)	DTI	N/A	No significant difference in long-range connectivity ↓ Local connectivity	Local connectivity defined as fiber tracts within the frontal lobe (spanning 35 voxels). Long-distance connectivity defined as fibers projecting from frontal lobe to other brain regions.
High functioning ASD (n = 15; mean age: 10.8 years) (Sundaram and others 2008)	Executive function task MEG	↑ Mistakes in ASD group in executive function task. ↑ Perseverative errors in ASD	↓ Long-range connectivity/synchrony ↑ Local connectivity/synchrony	Local connectivity defined by intraparietal synchrony. Long-distance connectivity defined by synchrony between the frontal-parietal networks.

ACC = anterior cingulate cortex; ADOS = Autism Diagnostic Observation Schedule; DTI = diffusion tensor imaging; EEG = electroencephalography; FFA = fusiform face area; fMRI = functional magnetic resonance imaging; IFG = inferior frontal gyrus; MEG = magnetoencephalography; PDD-NOS = pervasive-developmental disorder not otherwise specified; PCC = posterior cingulate cortex.

Functional connectivity can be assessed with most commonly available neuroimaging technologies, however, there are currently no strong models that could explain the behavioral patterns observed in ASD at the neuronal circuit level. Nevertheless, it is commonly, although not universally agreed, that anomalies in the interplay between long-range (including interlobe) and short-range (region-specific) connectivity relate to the behavioral theory of weak central coherence that, to some degree, explains both the social impairments and the superior performance in certain tasks of sensory perception (Menassa and others 2018). Accordingly, this assumes that higher order, social processes are reliant on intact large-scale connectivity, whereas putatively low-level perception can be accomplished with predominantly local circuitry.

**Figure 3.**
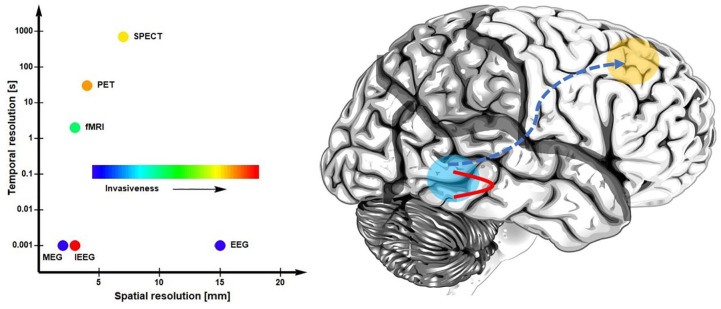
Connectivity in autism spectrum disorders (ASD). Left: spatial and temporal resolutions of common neuroimaging technologies used in measuring connectivity in ASD. Right: an illustration of the putative anomalies in functional connectivity in ASD, where local connections are favored over long-range interactions. MEG, magnetoencephalography; EEG, electroencephalography; fMRI, functional magnetic resonance imaging; PET, positron emission tomography; SPECT, single-photon emission computed tomography; IEEG, intracranial electric recordings.

The first indications that long-range functional connectivity is impaired in ASD date back to the late 1980s, when positron emission tomography was used to show reduced correlations in glucose metabolism between frontal cortices and other brain areas in resting adults with ASD (Horwitz and others 1988). Subsequently, functional magnetic resonance imaging (fMRI) has been used, where connectivity is typically defined in terms of correlation coefficients between regional BOLD (blood oxygen level–dependent) time-series, or spatial coherence patterns. Indeed, such studies have revealed impaired connectivity both within the frontal lobe and between frontal and temporal cortices during rest and a variety of task conditions, such as, face recognition (Koshino and others 2008), sentence comprehension (Just and others 2004) and the processing of emotional expressions (Sato and others 2012). Such findings, augmented by computational models of executive functioning, have led to the theory of frontal-posterior underconnectivity in ASD (Just and others 2012). Moreover, a recent fMRI study suggests that ASD individuals show reduced functional connectivity between the hippocampus and regions of the frontal-parietal network (Cooper and others 2017), implying that underconnectivity might involve non-neocortex and perhaps subcortical structures (Fig. 3).

Interestingly, underconnectivity in ASD compared to typically developing subjects can be observed at the whole-brain level, as well as locally in visual networks (Moseley and others 2015), where effects are intermediate in relatives who share some behavioral patterns with their affected siblings. This suggests that impairments in connectivity are heritable to some degree. The putative clinical relevance of functional connectivity is further supported by findings from a longitudinal study suggesting that anomalies default-mode network and frontal-parietal task control network correlate with future ASD traits and changes in adaptive behaviors (Plitt and others 2015).

Although underconnectivity has been observed often, this phenomenon might not always be present as suggested by a large-scale study in ASD (Woodward and others 2017). Using data culled from the Autism Brain Imaging Data Exchange, the authors examined thalamocortical functional connectivity as quantified by resting fMRI. The results suggest the prefrontal cortex, temporal cortex, and sensorimotor cortex show increased (hyper-) connectivity with the thalamus in individuals with ASD but not in typically developing subjects. The associations found between connectivity patterns and clinical symptoms, however, were not significant, making it impossible to judge the clinical relevance of observed hyper-connectivity. The notion of thalamocortical hyper-connectivity was recently corroborated using resting fMRI data obtained in adult males with ASD (Iidaka and others 2019). Interestingly, authors found some evidence that the pathophysiology of ASD is more likely related to thalamocortical hyper-connectivity than to amygdala-cortical hypo-connectivity, which has been observed in the past in line with the underconnectivity account of autism. Noteworthy, hyper-connectivity (assessed with resting fMRI) between the thalamus and cortical regions was found children and adolescents with ASD relative to typically developing children (Mash and others 2020).

In studies employing electroencephalography (EEG) and magnetoencephalography (MEG), functional connectivity is typically defined in terms of a correlation of signals in frequency bands commonly known as fundamental rhythms, which have been robustly linked to a large number of cognitive processes and brain states. The precise neuronal mechanism underlying the rhythms are still elusive, although there is evidence that higher frequency oscillations emerge from the coordinated interaction of inhibition and excitation (Buzsáki and Wang 2012). Broadly in line with fMRI findings, resting-state EEG studies of ASD have reported reduced coherence between frontal and occipital regions for delta (1-2 Hz) and theta (3-7 Hz) bands (Coben and others 2008) and reduced connectivity between the frontal cortex and the temporal and parietal cortices for the alpha (8-12 Hz) band (Murias and others 2007).

There is some evidence for increased short-range frontal connectivity in the delta band (Barttfeld and others 2011) and increased local connectivity in occipital cortices (Berman and others 2015) as evidenced by a measure known as alpha-to-gamma (>30 Hz) phase-amplitude coupling (PAC). Using a measure of evoked (i.e., stimulus-locked) gamma oscillation detected with MEG, increased 40 Hz coherence following semantically incongruous sentences was observed over frontal regions (Braeutigam and others 2008) whereas increased frontal-temporal functional connectivity in ASD was observed in a perceptual discrimination task (Menassa and others 2018). In contrast, a form of generalized underconnectivity has been reported in ASD for face processing tasks using coherence and PAC measures applied to EEG recordings (Khan and others 2013). It appears that the picture emerging from electrophysiological studies is at least as complex as the one suggested by fMRI, where in addition to underconnectivity, aberrant over-coherence might have a greater etiological relevance than previously assumed. This view would be supported by a recent study that functional whole-brain connectivity in the theta band at 14 months correlates with severity of restricted behaviors at 36 months in infants who met criteria for ASD (Haartsen and others 2019).

Considering the roles of the medial prefrontal cortex (area 32) and anterior cingulate cortex, the long cortico-cortical connection with the precuneate parietal cortex may be of special interest. Associative pathways connecting these areas form the backbone of the structural connectome and these areas are known to have functions of self-awareness and social cognition. Synaptic connectivity of these regions shows earlier establishment of long-range connectivity than lateral neocortical areas. Here, the timing and differential vulnerability of abundant presynaptic input to these medial prefrontal cortical areas may be one of the multiple routes of abnormal synaptic connectivity.

Taken together, there is growing evidence that ASD is associated with altered patterns of functional and brain connectivity (see also O’Reilly and others, 2017 for a recent review of electrophysiological studies). In addition to the observations discussed above, further studies are listed in Table 3, which also points to relevant observations based on diffusion tensor imaging (DTI) as a bridge between anatomical and functional connectivity. Specifically, long-range connectivity appears generally impaired, that is, reduced, compared with typically developing subjects. However, this might not hold under certain task conditions. The case of short-range connectivity is less clear, implying that the coexistence of impaired, unimpaired, or possibly enhanced skills is not simply explained in terms of network scale. Although definitive answers are not readily available, we are beginning to better understand the relationship between functional and synaptic-structural connectivity, which will help to assess the meaning and clinical relevance of functional connectivity. Indeed, a recent review of combined (resting state) fMRI and DTI found a significant quantitative structure-function relationship suggesting that anatomical connectivity provides the basis from which functional connectivity emerges (Straathof and others 2019).

## Altered Energetic States in ASD May Explain Altered Connectivity

The wiring economy principle dictates that the metabolic costs of functionally resourcing a brain are large and governs its connectivity structure (Laughlin and Sejnowski 2003; Raj and Chen 2011). Given that the cost of wiring the brain may scale as the square of the wire length (Chklovskii 2004), it may be hypothesized that altered developmental connectivity in ASD may imbalance wiring cost optimization. This may be met by insufficient resource allocation and reduced development of more “costly” long-distance connections, resulting in a situation of local overconnectivity and distal underconnectivity.

A putative direct mechanism underlying distal underconnectivity may result from the inherent vulnerability of long-distance connections to glutamate and oxidative stress. Although there is a distinct lack of literature investigating energetic states in long-distance/interlobar connections, a parallel may be drawn with long, highly arborized dopaminergic neurons from the substantia nigra. For example, the energy cost of axonal action potential generation and membrane recovery increases with the size and complexity of the axonal arbor of such dopaminergic neurons (Pissadaki and Bolam 2013).

## The Imbalance between Excitation and Inhibition Is Core to ASD Pathophysiology

With the emergence of computer modelling approaches of neural networks, the dynamic effects of clustered and overconnected circuits can be investigated. Although current models may lack the necessary complexity to model the possible heterogeneity of individual neuron response profiles, they have generally shown that a rewiring of only 3% of excitatory connections can substantially change balanced network dynamics (Litwin-Kumar and Doiron 2012).

In 2003, Rubenstein proposed that inherent to some forms of ASD is an increased cortical excitation to inhibition ratio (E/I), resulting in hyperexcitability of cortical circuits (Rubenstein and Merzenich 2003). Over a decade later, this theory has been bolstered by various studies (Robertson and others 2016; Sohal and others 2009; Wilson and others 2007) and is consistent with the observed prevalence of epilepsy in ASD that is some 25 times the rate found in the general population (Bolton and others 2011; Bozzi and others 2018). Why glutamatergic/excitatory and GABAergic/inhibitory networks and transmission may be differentially affected in ASD remains an open question. In a recent critical literature review, abnormal GABAergic and glutamatergic neurotransmission in key brain areas have both been implicated in E/I imbalance in ASD (Uzunova and others 2016). This may reflect the outcome of asymmetry in E/I ratios across different cortical networks that is possibly linked to their relative composition of interneuron subtypes, which show heterogeneity in E/I synaptic inputs (Gulyás and others 1999). Network E/I balance in certain brain regions may therefore be differentially affected by, or themselves affect, network overconnectivity. Future studies may benefit from investigating functional patterns of E/I ratios across cortical areas. This may provide further insight into the possible susceptibility of certain cortical networks to E/I imbalance that may result from, or possibly in, ASD-linked overconnectivity.

## Vulnerability of Frontal Networks in ASD and Phenotypic Specificity

A further theory linked to ASD involves the early closure of neuroplastic critical windows (Berger and others 2013). The idea that a precise balance of E/I transmission may be required for critical window plasticity (LeBlanc and Fagiolini 2011), which otherwise would be compromised if the E/I balance is offset, may provide a means to unify ASD theories. Given that the development of frontal networks may strongly depend on experience-dependent input during a critical plasticity window (age 2-3 years), premature closure of such a window may further compound the susceptibility of frontal networks in ASD.

In fact, stimulated elevation of the E/I ratio in the mouse PFC via optogenetic approaches, elicits a profound impairment in cellular information processing and has been associated with autistic-like behaviors (Yizhar and others 2011). These findings may accredit a “two-hit” mechanism in which E/I imbalance affects frontal networks, first at the level of the developmental critical period, and second at the level of circuit dynamics and network function following this. Networks less dependent on such critical periods would be less affected by the first “hit” and may be relatively spared in ASD. Such a mechanism may therefore account for the network and phenotypic specificity in ASD (Table 4).

**Table 4.** Involvement of Frontal Networks in Autistic Behaviors.

Reference	Method	Behavior	Findings
(Abbott and others 2018)	fMRI study examining functional connectivity of frontal systems in 50 ASD vs. 52 TD controls	Repetitive behaviors	↓ Frontoparietal/limbic circuit ratios correlated with ↑ repetitive behaviors in ASD group
(Thakkar and others 2008)	fMRI study investigating ACC activation to correct and erroneous anti-saccades in 10 ASD vs 14 TD. DTI assessed ACC white matter integrity	Repetitive behaviors	↑ ACC activation on correct anti-saccade was correlated to ↑ Repetitive behaviors in ASD group
(Pinkham and others 2008)	fMRI study to explore the neural activation of discrete brain regions during complex social judgement of faces task in 12 ASD vs 12 TD controls	Impaired social functioning	↓ Neural activation in right amygdala, fusiform face area and left ventrolateral PFC in ASD group

ACC = anterior cingulate cortex; ASD = autistic spectrum disorders; fMRI = functional magnetic resonance imaging; PFC = prefrontal cortex; TD = typically developing.

It is also possible that the specificity of circuits most adversely affected in ASD may be governed by their relative dependency on experience-dependent plasticity for development—reflected by the subsequent differences in histogenesis duration between functional areas that may otherwise be disturbed in ASD (Doll and Broadie 2014; Ebert and Greenberg 2013).

Frontal brain networks show the most protracted development out of all brain regions (Huttenlocher and Dabholkar 1997; Schneider and others 2004; Sousa and others 2018), presumably alluding to the developmental dependence of these regions and subsequent development of complex cognitive, social and behavioral abilities on life experience. Several lines of evidence implicate the dependence of normal frontal network development and function on experience-dependent plasticity (Bock and others 2008; Kolb and others 2012). This can be contrasted to other circuit functions, that are relatively spared in ASD (Kéïta and others 2010; Shafai and others 2015). For example, visual orientation discrimination thresholds are not different between individuals with ASD and healthy controls (Shafai and others 2015). Given that orientation selectivity can still develop, albeit to a reduced level, in visually deprived animals (Chapman and Stryker 1993), it may be suggested that circuits relatively spared in ASD are ones that show a greater degree of intrinsic functional hardwiring. Such circuit functionality may therefore be less dependent on extrinsic, experience-dependent plasticity mechanisms.

## Conclusion

Despite the genetic heterogeneity in ASD, two key biological themes, namely synaptic non-plasticity and abnormal brain connectivity, link idiopathic and syndromic ASDs at the level of altered biological function. However, the manner in which ASD-linked synaptic non-plasticity might lead to specific patterns of local overconnectivity, distal underconnectivity, and phenotypic specificity has not yet been solved. The inherent dependence of frontal brain networks on experience for normal development may underlie their vulnerability to disruption by ASD-linked synaptic non-plasticity. The “two-hit” model proposed here, in which brain overconnectivity may disrupt E/I balance, and the critical developmental period needed for the development of frontal networks, as well as normal network dynamics and function, may further compound frontal network vulnerability. This may explain phenotypic specificity in some cases of ASD. Connectivity patterns in ASD may arise due to impingement of ASD-synaptic mutations on microglial pruning functions. This model may inform novel therapeutic strategies aimed at rescuing cellular functions of synaptic plasticity and may provide insight into the etiology of ASD.
